# C1GALT1 Promotes Invasive Phenotypes of Hepatocellular Carcinoma Cells by Modulating Integrin β1 Glycosylation and Activity

**DOI:** 10.1371/journal.pone.0094995

**Published:** 2014-08-04

**Authors:** Chiung-Hui Liu, Rey-Heng Hu, Miao-Juei Huang, I-Rue Lai, Chia-Hua Chen, Hong-Shiee Lai, Yao-Ming Wu, Min-Chuan Huang

**Affiliations:** 1 Graduate Institute of Anatomy and Cell Biology, National Taiwan University College of Medicine, Taipei, Taiwan; 2 Department of Surgery, National Taiwan University Hospital, Taipei, Taiwan; 3 Research Center for Developmental Biology and Regenerative Medicine, National Taiwan University, Taipei, Taiwan; Institute of Hepatology - Birkbeck, University of London, United Kingdom

## Abstract

Cancer cell invasion and metastasis are the primary causes of treatment failure and death in hepatocellular carcinoma (HCC). We previously reported that core 1 β1,3-galactosyltransferase (C1GALT1) is frequently overexpressed in HCC tumors and its expression is associated with advanced tumor stage, metastasis, and poor survival. However, the underlying mechanisms of C1GALT1 in HCC malignancy remain unclear. In this study, we found that overexpression of C1GALT1 enhanced HCC cell adhesion to extracellular matrix (ECM) proteins, migration, and invasion, whereas RNAi-mediated knockdown of C1GALT1 suppressed these phenotypes. The promoting effect of C1GALT1 on the metastasis of HCC cells was demonstrated in a mouse xenograft model. Mechanistic investigations showed that the C1GALT1-enhanced phenotypic changes in HCC cells were significantly suppressed by anti-integrin β1 blocking antibody. Moreover, C1GALT1 was able to modify O-glycans on integrin β1 and regulate integrin β1 activity as well as its downstream signaling. These results suggest that C1GALT1 could enhance HCC invasiveness through integrin β1 and provide novel insights into the roles of O-glycosylation in HCC metastasis.

## Introduction

Hepatocellular carcinoma (HCC) is one of the most aggressive tumors making it the third leading cancer-caused deaths worldwide [1]. A majority of HCC treatment failures arise from vascular invasion, metastasis, and recurrence after surgical resection [2]–[4]. Similar to other cancers, HCC metastasis is a multistep process that involves tumor cell proliferation, invasion, dissemination, immune evasion, and growth at distal sites [5], [6]. Alterations in cancer cell and extracellular matrix (ECM) interactions in the tumor microenvironment are essential to initiate the process of metastasis [7]–[10]._ENREF_7 Recent studies have highlighted the importance of glycosyltransferases in regulating cell-ECM interactions through modulation of integrin functions [11]–[14]. However, the contributions of O-glycosylation in the interaction between HCC cells and the ECM have long been overlooked in the past.

Mucin-type O-glycosylation is the most common type of O-glycosylation, and it modulates diverse functions of membrane-bound and secreted proteins [15]. Mucin-type O-glycans are formed when N-acetylgalactosamine (GalNAc) is added to a serine (Ser) or threonine (Thr) residue to form a GalNAcα1-Ser/Thr structure (Tn antigen). Core 1 β1,3-galactosyltransferase (C1GALT1) transfers galactose (Gal) to the Tn antigen forming the Galβ1-3GalNAcα1-Ser/Thr structure (T antigen; core 1 structure). The T antigen is a precursor for subsequent elongation of mucin-type O-glycans [16]. In normal tissues, complex O-glycan structures are formed through a series of glycosyltransferases in the Golgi apparatus [15]. In cancer cells, however, short mucin-type O-glycans are often abnormally present on cell surfaces. Expression of the short O-glycans positively correlates with tumor malignancy in many types of cancer [17]–[20]. Although this correlation has been established, however, the biological functions of the short O-glycans in cancer cells and their influence on protein functions are largely unclear.

We recently reported that up-regulation of C1GALT1 modulates O-glycosylation in HCC cells and C1GALT1 expression is associated with advanced tumor stage, metastasis, and poor prognosis in HCC [21]. We also reported that C1GALT1 enhances HGF-triggered cell proliferation through MET, however, the detailed mechanisms by which C1GALT1 mediates cell invasion and metastasis are unclear. In this study, we investigated the effects of C1GALT1 on HCC cell invasive behaviors and the underlying mechanisms.

## Materials and Methods

### Ethics Statement

All animal experiments in this study were reviewed and approved by the Institutional Animal Care and Use Committee (IACUC) of National Taiwan University College of Medicine.

### Reagents and antibodies

Antibodies against total integrin β1 and activated integrin β1 (HUTS-21) were purchased from BD Biosciences (Lincoln Park, NJ). Integrin β1 blocking antibody (P4C10) was purchased from Millipore (Billerica, MA). Antibodies against C1GALT1, GAPDH, and focal adhesion kinase (FAK) were purchased from Santa Cruz Biotechnology, Inc. (Santa Cruz, CA). Antibody against phospho (p)-FAK was purchased from Cell Signaling Technology Inc. (Beverly, MA). Antibody against actin was purchased from GeneTex Inc. (Irvine, CA). *Vicia villosa* agglutinin (VVA) and peanut agglutinin (PNA) lectins were purchased from Vector Laboratories (Burlingame, CA). Human collagen IV, human fibronectin, murine laminin, bovine serum albumin (BSA), and protein de-glycosylation kit were purchased from Sigma (St Louis, MO).

### Cell culture

Human liver cancer cell lines, Sk-Hep1 and HepG2, were purchased from Bioresource Collection and Research Center (Hsinchu, Taiwan) in year 2008. HA22T and HCC36 cells were kindly provided by Prof. Shiou-Hwei Yeh (National Taiwan University) in year 2010. All cell lines were cultured in DMEM containing 10% fetal bovine serum (FBS) in 5% CO_2_ at 37°C.

### Transfection and RNA interference

Transfection and RNA interference were carried out as previous description [21]. To establish stable transfectants, pcDNA3.1/*C1GALT1* plasmid- and empty pcDNA3.1 plasmid-transfected HCC36 cells were selected with 600 µg/ml of G418 for 14 days. Stable clones were pooled together for further studies. The pLKO/*C1GALT1*-shRNA plasmid and non-targeting pLKO plasmid transfected HA22T cells were selected with 500 ng/ml of puromycin for 10 days. Stable C1GALT1 silencing clones were examined by Western blotting.

### Western blotting

Western blotting was performed as reported previously [20].

### Cell migration and invasion assay

Assays were performed in BioCoat transwell chambers (BD Pharmingen) with uncoated porous filters (pore size 8 µm) to evaluate cell migration, or Matrigel coated porous filters to exam cell invasion. Cells were starved in serum free DMEM for 5 h before experiments. HCC cells (2×10^5^) in 0.2 ml serum-free DMEM were seeded into upper part of the chamber, and 0.6 ml DMEM containing 10% FBS was loaded in lower part of the chamber. For integrin β1 blocking experiments, cells were incubated with integrin β1 blocking antibody at room temperature for 10 min, and then seeded into chambers. Non-specific mouse IgG served as an isotype control. Cells in chambers were removed after 24 h incubation. Filters were fixed in methanol and stained with 0.1% crystal violet (Sigma) for counting. Independent experiments were repeated at least three times. Values for cell migration or invasion are expressed as the average number of cells per microscopic field over three fields per one filter.

### Cell adhesion assay

96 wells plates were coated with collagen IV, fibronectin, laminin, or BSA at concentrations of 5 µg/ml in PBS for 16 h, and then blocked with 1% BSA in PBS at 37°C for 2 h. HCC cells were dettached by 10 mM EDTA and 2×10^4^ cells in 100 µl serum-free DMEM per well were allowed to attach on coated plates at 37°C for 1 h. For integrin β1 functional blocking experiments, antibody and mouse IgG were treated for 10 min before seeding to coated plates. Attached cell number was counted manually under an inverted microscope and averaged from three independent experiments. For Western blotting, the experiments were carried out in 6 well plates (2×10^5^ per well), and cells were collected and lysed for analysis.

### Experimental metastasis model in NOD/SCID mice

Female NOD/SCID mice, 6–7 weeks of age, were purchased from National Laboratory Animal Center (Tainan, Taiwan). HA22T or HCC36 stable transfectants were injected into tail veins of mice (1×10^6^ cells/mouse). Mice were sacrificed 8 weeks after inoculation. The lungs were excised and surface nodules on lungs of each mouse were counted. Excised tissues were paraffin-embedded for hematoxylin and eosin (H&E) staining and immunohistochemistry.

### Flow cytometry

Cell surface protein or carbohydrate expression was analyzed using FACScan cytometer (BD Pharmingen). HCC cells were detached with 10 mM EDTA and resuspended in PBS with 1% BSA. Cells (1×10^5^/100 µl) were incubated with primary antibodies at 1∶100 dilutions on ice for 30 minutes. Cells were washed twice with ice cold 1% BSA/PBS and then incubated with FITC-conjugated secondary antibody on ice for 30 min. The fluorescence intensity of 1×10^4^ cells for each sample was analyzed. Mouse IgG was used as isotype control. The mean florescence intensity (MFI) was averaged from three independent experiments.

### De-glycosylation, lectin pull down, and immunoprecipitation

Protein de-glycosylation was carried out as previously described [21]. For lectin pull down assay, cell lysates (0.5 mg) were applied to PNA conjugated agarose beads at 4°C for 16 h. For immunoprecipitation assay, cell lysates (1.2 mg) were incubated with 5 µg of anti-integrin β1 antibody and protein G sepharose beads (Amersham Pharmacia) at 4°C for 16 h. The pull-downed proteins were then analyzed by Western blotting.

### Statistics

All data analysis was performed using GraphPad Prism 5.01. An analysis of variance (ANOVA) and Student's t test were used for comparison among groups. Bar graphs represent means ± SD. *P*<0.05 was considered significant.

## Results

### C1GALT1 promotes the adhesion, migration, and, invasion of HCC cells

**Figure 1:**
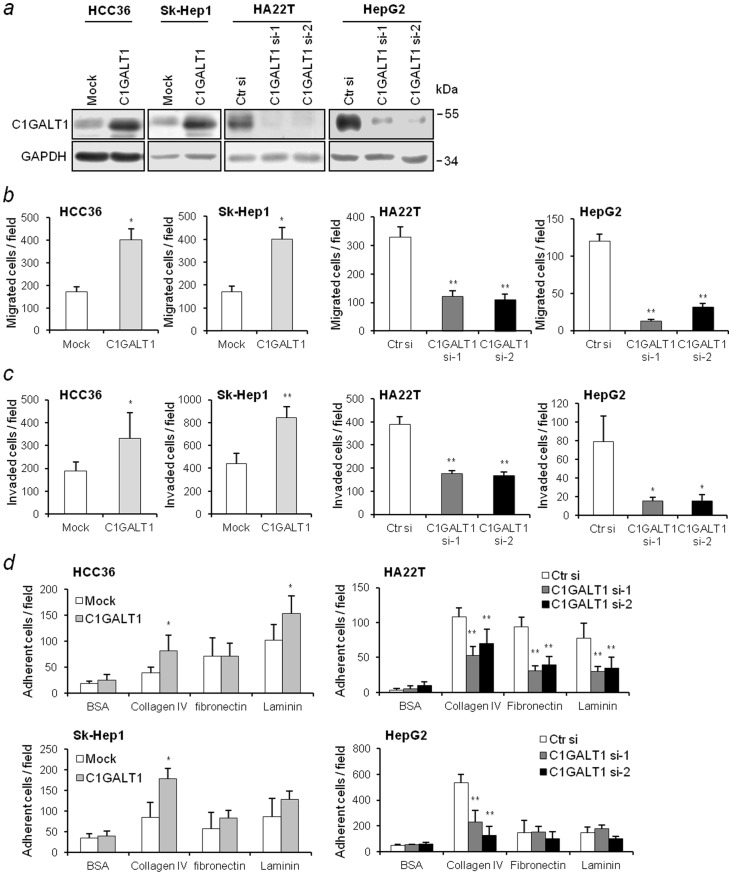
C1GALT1 regulates cell adhesion, migration, and invasion. (*a*) Overexpression and knockdown of C1GALT1 in HCC cell lines. Overexpression of C1GALT1 with *C1GALT1*/pcDNA3.1 plasmid compared with pcDNA3.1 empty plasmid (mock) transfection in HCC36 and Sk-Hep1 cells. Knockdown of C1GALT1 with two *C1GALT1* siRNAs compared with control (Ctr) siRNA in HA22T cells and HepG2 cells. GAPDH was used as a loading control. (*b*) C1GALT1 regulated transwell cell migration and (*c*) Matrigel invasion. Overexpression of C1GALT1 significantly enhanced migration and invasion in HCC36 and HA22T cells (left), and knockdown of C1GALT1 suppressed migration and invasion in HA22T and HepG2 cells (right). All results are represented as means ± SD from three independent experiments. * *P*<0.05; **P<0.01. (*d*) C1GALT1 regulated cell adhesion. C1GALT1 overexpressed (left) or knockdown (right) cells were allowed to attach on collagen IV-, fibronectin-, and laminin-coated plates. Adherent cells were counted. BSA was used as a non-ECM protein control. Results are represented as the means ± SD from three independent experiments. * *P*<0.05; **P<0.01.

To investigate the effects of C1GALT1 on cell motility and invasiveness, C1GALT1 was overexpressed in Sk-Hep1 and HCC36 cells, that have low endogenous levels of C1GALT1, and C1GALT1 was knocked down with two different C1GALT1-specific siRNAs in HA22T and HepG2 cells, that express high levels of C1GALT1 (Fig. 1*a*). We found that the overexpression of C1GALT1 enhanced cell migration and invasion. Conversely, knockdown of C1GALT1 inhibited cell migration and invasion (Fig. 1*b and 1c*). As cell-ECM interaction is the initial step for cancer cell movement and metastasis [5], we investigated whether C1GALT1 regulates cell adhesion to ECM proteins. We found that the overexpression of C1GALT1 in HCC36 cells enhanced cell adhesion to collagen IV and laminin, and increased Sk-Hep1 cell adhesion to collagen IV. In addition, the knockdown of C1GALT1 by siRNA suppressed HA22T cell adhesion to collagen IV, fibronectin, and laminin, and inhibited HepG2 cell adhesion to collagen IV (Fig. 1*d*). Collectively, these results suggest that C1GALT1 can promote cell-collagen IV adhesion, migration, and invasion of HCC cells.

### C1GALT1 regulates HCC cell metastasis in NOD/SCID mice

**Figure 2:**
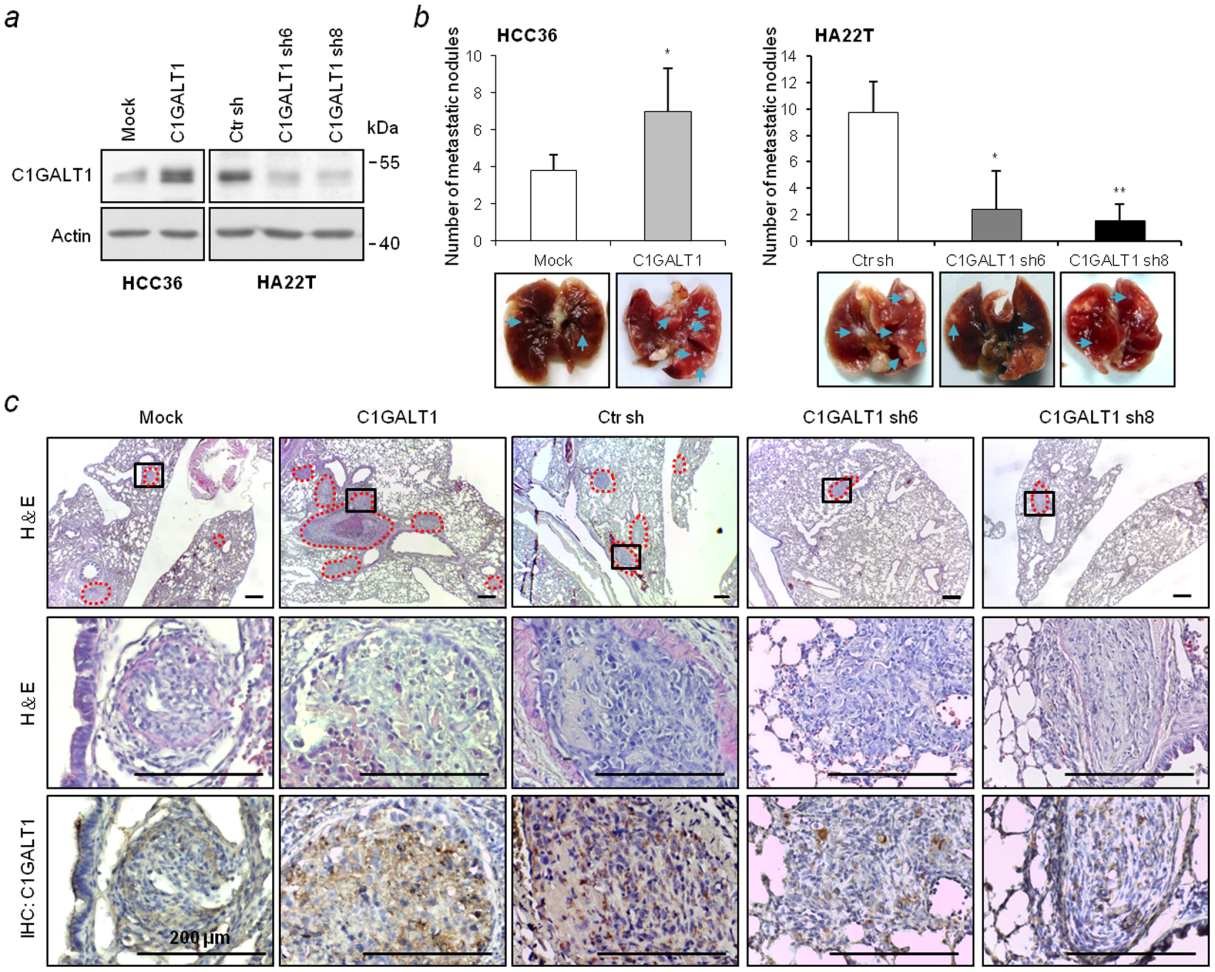
C1GALT1 regulates lung metastasis of HCC cells in NOD/SCID mice. (*a*) Stable overexpression and knockdown of C1GALT1 in HCC cells. The protein expression levels of C1GALT1 were analyzed by Western blotting. (*b*) Effects of C1GALT1 on lung metastasis. Metastatic tumors were increased in the C1GALT1 overexpressed group (left) and decreased in the C1GALT1 knockdown groups (right). Representative image of the excised lungs are shown at the bottom, n = 6 for each group. Results are shown as means ± SD. * *P*<0.05; **P<0.01. Blue arrows indicate the location of the tumor nodules on the lung surface. (*c*) H&E staining and immunohistochemistry of paraffin-embedded lung sections. Representative images (upper) and amplified images (middle) are shown. Immunostaining revealed the expression of C1GALT1 in metastatic tumors (bottom). The red dash line indicates the location of metastatic tumors. Scale bars  = 200 µm.

To analyze the effects of C1GALT1 on HCC cell metastasis *in vivo*, a mouse tail vein injection model was used. C1GALT1 was stably overexpressed in HCC36 cells and knocked down in HA22T cells (Fig. 2*a*). To measure the carbohydrate changes on the stable transfectants, flow cytometry with FITC-conjugated PNA was performed [22], [23]. Overexpression of C1GALT1 increased PNA binding to the surface of HCC cells, whereas knockdown of C1GALT1 decreased PNA binding (Figure S1), indicating that C1GALT1 catalyzes the formation of T antigen on the cell surface. The stable clones were injected into NOD/SCID mice, which were sacrificed after 8 weeks. The results showed that overexpression of C1GALT1 significantly enhanced the number of lung metastatic tumors, while knockdown of C1GALT1 reduced the number of lung metastatic nodules (Fig. 2*b*). H&E staining confirmed the location of tumors in the lung sections, and immunohistochemical results confirmed the overexpression or knockdown of C1GALT1 in HCC metastatic tumor nodules (Fig. 2*c*). These results suggest that C1GALT1 enhances the metastatic potential of HCC cells *in vivo*.

### C1GALT1 induces HCC cell adhesion, migration and invasion through integrin β1

**Figure 3:**
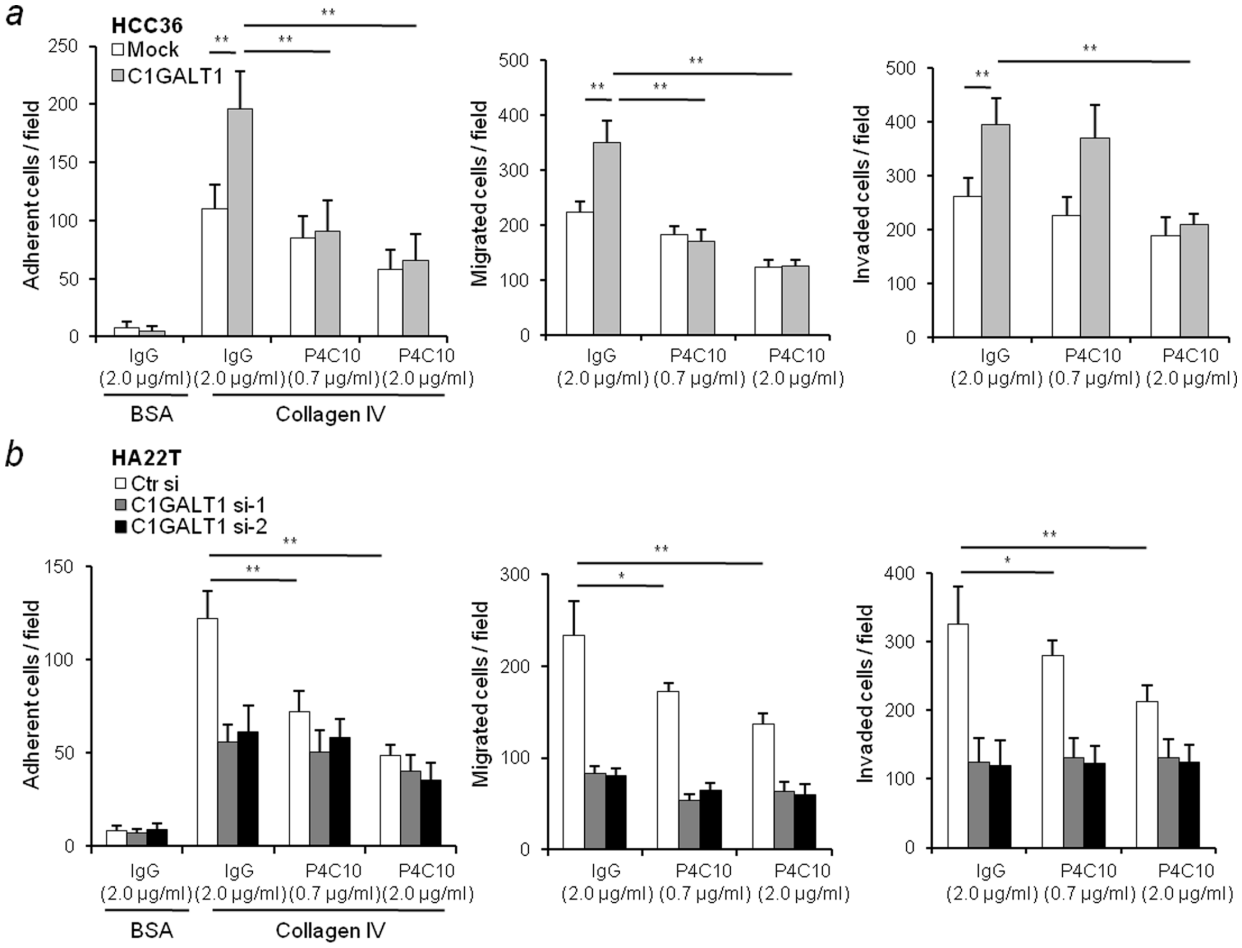
C1GALT1 induces HCC cell adhesion, migration and invasion through integrin β1. (*a*) The effects of the integrin β1-blocking antibody, P4C10, on C1GALT1 overexpressing cells. HCC cells were pre-treated with the indicated concentration of P4C10 or control IgG for 10 min. C1GALT1-induced cell-collagen IV adhesion (left), migration (middle), and invasion (right) were suppressed by the integrin β1-blocking antibody. Results are represented as means ± SD from three independent experiments. **P<0.01. (*b*) The effects of the integrin β1-blocking antibody on C1GALT1 knockdown cells. P4C10 inhibited cell-collagen IV adhesion (left), migration (middle), and invasion (right) in cells transfected with control siRNA (Ctr si). Results are shown as means ± SD. * *P*<0.05; **P<0.01.

The ECM receptors, such as integrins, govern cell-ECM interactions and are considered as crucial mediators of HCC cell invasion [24]–[26]. To test whether integrins are involved in the C1GALT1-mediated phenotypes, we first analyzed the mRNA expression of the integrin family in HCC cell lines. The results showed that the expression of integrin alpha subunits varied, while integrin β1 was the most commonly expressed beta subunit in all tested HCC cell lines (Figure S2). Interestingly, integrin β1 was reported to be an O-glycosylated protein [27]–[29]. To determine whether integrin β1 participates in C1GALT1-mediated cell-collagen IV adhesion, migration, and invasion, we used an integrin β1-blocking antibody, P4C10. The results showed that integrin β1 blockage in C1GALT1 overexpressing cells significantly suppressed C1GALT1-induced cell adhesion, migration, and invasion (Fig. 3*a*). Furthermore, in C1GALT1 knockdown cells, these phenotypes were not further suppressed by integrin β1-blocking antibody (Fig. 3*b*). These results suggest that C1GALT1 may regulate the invasive phenotypes of HCC cells by modulating integrin β1 signaling.

### C1GALT1 modulates integrin β1 glycosylation and activity

**Figure 4:**
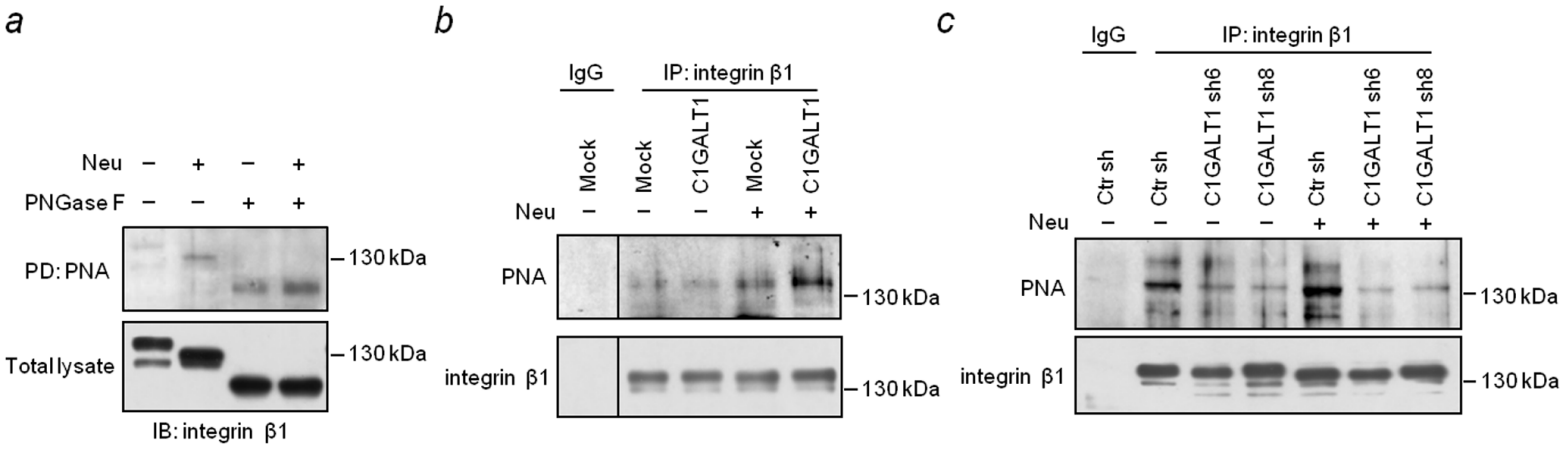
C1GALT1 modifies O-glycans on integrin β1. (*a*) Integrin β1 carried O-glycans in HCC cells. Lysates of HA22T cells (0.5 mg) were treated with neuraminidase (Neu) and/or PNGaseF and then pulled down (PD) by PNA agarose beads. The pulled down proteins were separated by 6% SDS-PAGE and analyzed by immunoblotting (IB) with anti-integrin β1 antibody. (*b*) C1GALT1 enhanced PNA binding to integrin β1 in HCC36 cells. (*c*) Knockdown of C1GALT1 suppressed PNA binding to integrin β1 in HA22T cells. Cell lysates (1.2 mg) were immunoprecipitated (IP) by anti-integrin β1 antibody, and were treated with (+) or without (−) neuraminidase. Proteins were separated by 8% SDS-PAGE, and then blotted with PNA or anti-integrin β1 antibody. Non-specific mouse IgG was used as control.

To investigate the underlying mechanisms of C1GALT1 in integrin β1 signaling, we analyzed the glycosylation and activity of integrin β1 in HCC cells. As the O-glycosylation of integrin β1 in HCC cells has never been reported, we first analyzed whether integrin β1 carries O-glycans in HCC cells. Lectin pull-down assays demonstrated that PNA could bind integrin β1 and the binding was further increased when sialic acids and N-glycans were removed by neuraminidase and PNGaseF, respectively (Fig. 4*a*). These results suggest that integrin β1 carries T and sialyl T antigens in HCC cells. To investigate effects of C1GALT1 on the O-glycan structures on integrin β1, immunoprecipitation with anti-integrin β1 antibody and PNA lectin blotting were performed. The results showed that the overexpression of C1GALT1 enhanced PNA binding to integrin β1 (Fig. 4*b*). Conversely, the knockdown of C1GALT1 decreased PNA binding to integrin β1 (Fig. 4*c*). We also observed that PNA binding to integrin β1 was further increased after neuraminidase treatment. These data strongly support that C1GALT1 expression modifies O-glycans on integrin β1 and increases the expression of T and sialyl T antigens.

**Figure 5:**
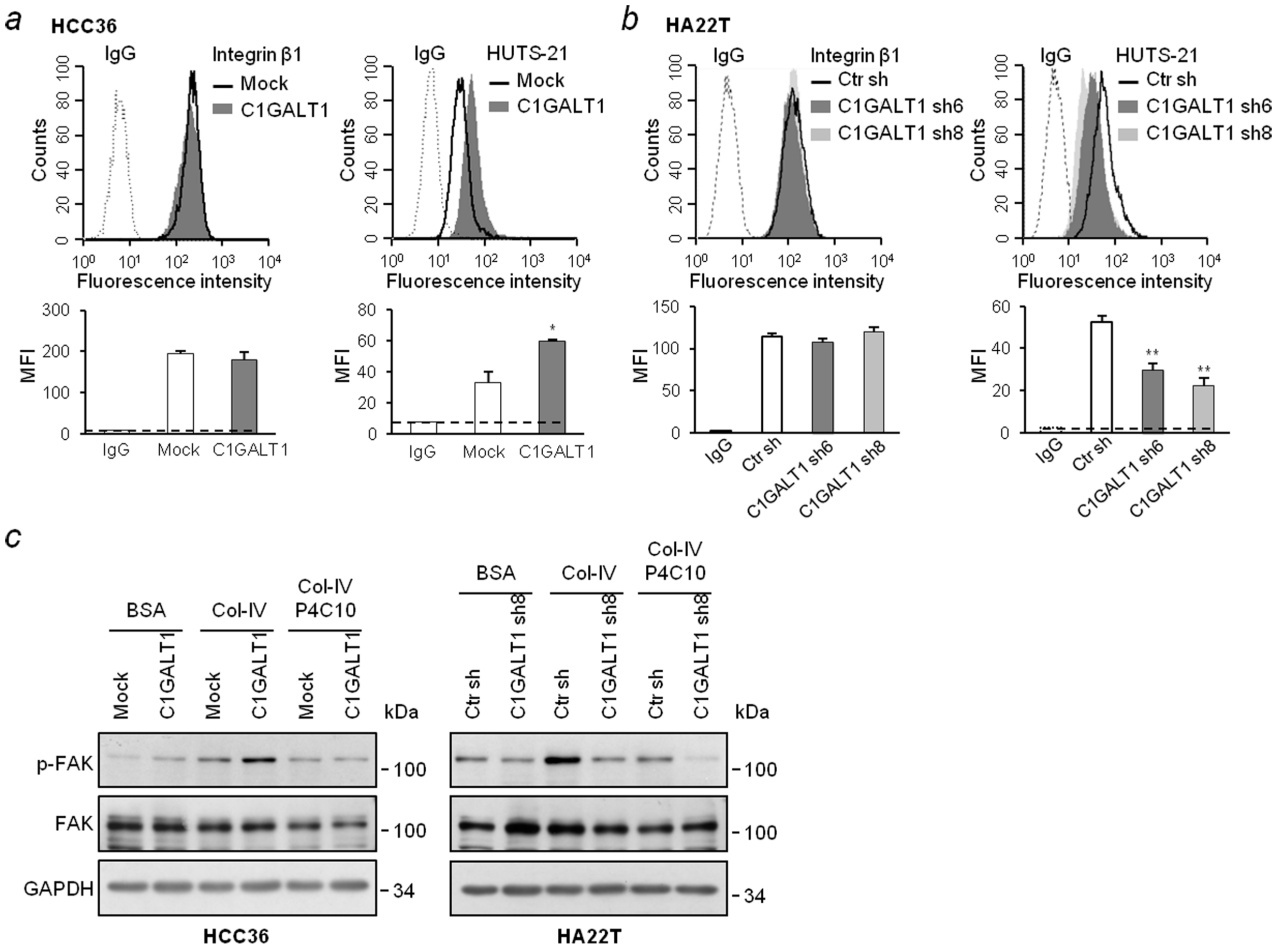
C1GALT1 regulates integrin β1 activity and signaling. (*a*) The activity of integrin β1 was enhanced in C1GALT1 overexpressing cells. Mock and C1GALT1 transfectants were analyzed by flow cytometry with anti-integrin β1 antibody (left) or anti-activated integrin β1 (HUTS-21) antibody (right). Non-specific mouse IgG was used as a background fluorescence control (dash line). The mean fluorescence intensities (MFI) are shown at the bottom. Results are represented as means ± SD from three independent experiments. * *P*<0.05. (*b*) Knockdown of C1GALT1 suppressed integrin β1 activity. Control (Ctr sh) and C1GALT1 knockdown (C1GALT1 sh6 and C1GALT1 sh8) cells were analyzed by flow cytometry with the indicated antibodies. The average of MFI is shown at the bottom. Results are represented as means ± SD from three independent experiments. * *P*<0.05. (*c*) C1GALT1 regulated integrin β1-induced FAK activation. p-FAK (Tyr397) and total FAK in C1GALT1 overexpressing cells (left) and knockdown cells (right) were analyzed by Western blotting. Transfectants were seeded on BSA or collagen IV (Col-IV)-coated plates for 0.5 h. The integrin β1 blocking antibody (P4C10, 2 µg/ml) was incubated with cells for 10 min before seeding on collagen IV-coated plates. GAPDH was used as a loading control.

To investigate whether changes in O-glycan alter the functions of integrin β1, flow cytometry with an activated integrin β1-specific antibody, HUTS-21, was performed [30], [31]. We found that C1GALT1 overexpression increased the expression of activated integrin β1 (Fig. 5*a*). Conversely, C1GALT1 knockdown decreased the expression of activated integrin β1 (Fig. 5*b*). The expression levels of total integrin β1 on cell surfaces were not changed (Fig. 5a & 5b, left panels). To further confirm that integrin β1 activity contributes to cell-collagen IV adhesion, integrin β1-blocking antibody was used and phosphorylation of FAK was analyzed. We found that overexpression of C1GALT1 enhanced adhesion-induced FAK activation in HCC36 cells; whereas knockdown of C1GALT1 suppressed adhesion-induced FAK activation in HA22T cells (Fig. 5*c*). In addition, FAK activation was inhibited by pre-treatment with the integrin β1-blocking antibody. Together, these data suggest that C1GALT1 modulates O-glycans on integrin β1 and regulates integrin β1 activation and its downstream signaling.

## Discussion

We previously found that overexpression of C1GALT1 is associated with HCC metastasis and poor survival, and we identified MET as one of the important acceptor substrates of C1GALT1 in regulating cell proliferation [21]. Here, we demonstrated that C1GALT1 increased HCC cell adhesion, migration, and invasion *in vitro*, and enhanced tumor metastasis *in vivo*. In addition, we found that C1GALT1 modified O-glycans on integrin β1 and regulated its activity as well as downstream FAK signaling in HCC cells. These results provide mechanistic insights into the contribution of C1GALT1 in HCC invasiveness and metastasis.

A recent glycoproteomic study identified O-glycosylation sites on integrin β1 [32]. Using immunoprecipitation and lectin blotting assays in this study, we found that T and sialyl T antigens on integrin β1 were modified by C1GALT1 in HCC cells. In primary HCC tissues, we also found the expression of these short O-glycans on integrin β1, as revealed by PNA lectin pull-down assay (data not shown). As the genetic background is different for each patient and HCC tumors, the presentation of short O-glycans could be affected by various factors, such as the expression of core 2 or core 1 extension enzymes and aberrant protein trafficking in cancer cells [17], [33]._ENREF_39 Our study provides evidence that C1GALT1 is one of the crucial factors that modify O-glycans on integrin β1 in HCC. It has been reported that sialyl Tn and Tn antigens modulate integrin β1 activity and its downstream signaling [28], [29]. Core 3 O-glycans on integrin β1 reduced its maturation in prostate carcinoma cells [27]. Here, we found that enrichment of T and sialyl T antigens on integrin β1 could enhance integrin β1 activity. These results not only suggest a novel role of T and sialyl T antigens in regulating integrin β1 activity but also suggest differential effects of O-glycan structures on integrin β1 activity. It has been reported that β1,4-galactosyltransferase 1 (B4GALT1) is highly expressed in HCC and can modify both N-glycans and core 2 O-glycans of glycoproteins [34], [35]. It will be of great interest to further investigate whether B4GALT1 can also modify the glycans on integrin β1 and regulate integrin β1 signaling.

It is well known that integrins have a great impact on cancer malignancy, making them appealing targets for cancer therapy [8], [9]. The αvβ3 and αvβ5 integrin inhibitor has shown therapeutic activity in glioblastoma clinical trials [36]. _ENREF_41 Blocking integrin β1 signaling can attenuate malignant phenotypes in breast cancer cells [37]. HCC cells with higher activities of integrin β1 have also been suggested to be more invasive [24], [25], [38]. Recent studies indicate that integrin β1 signaling is involved in ANGPTL4- and EPHA4-induced malignant phenotypes in HCC [39], [40]. However, the modulators that can promote integrin signaling remain unclear. In the present study, we found that overexpression of C1GALT1 increased integrin β1 activity, whereas the knockdown of C1GALT1 suppressed it in HCC cells. Previous reports suggest that FAK and p-FAK are overexpressed in HCC and their overexpression may contribute to the invasive behavior of HCC [41], [42]. In support of our previous results, we found that the phosphorylation level of FAK was increased in C1GALT1 overexpressing cells, whereas the p-FAK was decreased in C1GALT1 knockdown cells. These results suggest that C1GALT1 is a crucial regulator of integrin β1 signaling in HCC cells.

The present study and our previous work [21] underscore the importance of C1GALT1 in both integrin β1 and HGF/MET signaling pathways. It is not surprising that one O-glycosyltransferase could regulate various signaling pathways through different protein substrates [12], [20], [43], [44]._ENREF_24 In cancer cells, crosstalk between integrins and MET in regulating cell invasion, immunity, and development has been reported [45]–[48]._ENREF_42 Thus, it is reasonable to speculate that integrin β1 and MET signaling pathways cooperate to promote C1GALT1-mediated HCC malignancy, although detailed mechanisms remain to be further elucidated.

In conclusion, this study revealed a novel mechanism by which C1GALT1 promotes tumor metastasis. These findings further demonstrate the significant role of mucin-type O-glycosylation in modulating cancer malignancy and suggest that C1GALT1 may be a promising therapeutic candidate that targets multiple signaling pathways in HCC.

## Supporting Information

Figure S1 **C1GALT1 modulates O-glycan structures on cell surfaces of HCC cells.** (a) Binding specificity of PNA. Surface O-glycans on HCC36 cells were analyzed by flow cytometry with FITC-PNA. FITC-PNA was pre-incubated with lactose (1 mM), sucrose (1 mM), or solvent (H2O) for 15 minutes and then added to cells. The binding of PNA to cell surfaces was blocked by lactose, but not sucrose. The mean fluorescence intensities (MFI) are shown at the lower panel. Negative (-) indicates cells without adding FITC-PNA. (b) Effects of C1GALT1 overexpression on O-glycans in HCC36 cells. Overexpression of C1GALT1 enhanced PNA binding to HCC36 cells (left), and the signals were further increased after the removal of sialic acids by neuraminidase treatment (right). (c) Effects of C1GALT1 knockdown on O-glycans in HA22T cells. C1GALT1 knockdown inhibited PNA binding to HA22T cells. All results are represented as mean ± SD from three independent experiments. ** P<0.01
(PPTX)

Figure S2 **Expression of integrin family genes in HCC cell lines.** mRNA expression levels of integrin alpha subunits (*α 1* to *α 11* and *αV*) and beta subunits (*β 1* to *β 8*) in HCC cell lines were analyzed by real-time RT-PCR. The relative levels of *integrin* mRNAs were normalized to *GAPDH*.
(PPTX)
